# Metagenomic analysis of viral diversity and a novel astroviruse of forest rodent

**DOI:** 10.1186/s12985-022-01847-6

**Published:** 2022-08-31

**Authors:** Hai-chang Yin, De-cai Wan, Hong-yan Chen

**Affiliations:** 1 College of Life Science and Agriculture Forestry, Qiqihar University, Qiqihar, 161006 Heilongjiang China; 2 State Key Laboratory of Veterinary Biotechnology, Harbin Veterinary Research Institute, The Chinese Academy of Agriculture Sciences, 678 Haping Road, Harbin, 150069 China

**Keywords:** Metagenomics, Forest rodent, Virome, Novel, Astrovirus

## Abstract

**Background:**

Rodents are important virus reservoirs and natural hosts for multiple viruses. They are one of the wild animals that are extremely threatening to the spread of human viruses. Therefore, research on rodents carrying viruses and identifying new viruses that rodents carry is of great significance for preventing and controlling viral diseases.

**Methods:**

In this study, fecal samples from six species of forest rodents in Northeast China were sequenced using metagenomics, and an abundance of virome information was acquired. Selection of important zoonotic in individual rodents for further sequence and evolutionary analysis.

**Results:**

Among the top 10 most abundant viral families, RNA virus include Orthomyxoviridae, Picornaviridae, Bunyaviridae and Arenaviridae, DNA virus include Herpesviridae, Insect virus include Nodaviridae and Baculoviridae, Plant virus Tombusviridae and Phage (Myoriviridae). Except for Myoviridae, there was no significant difference in the abundance of virus families in the feces of each rodent species. In addition, a new strain of astrovirus was discovered, with an ORF and genome arrangement comparable to other rodent astroviruses.The newly identified astrovirus had the highest similarity with the rodent astrovirus isolate, CHN/100.

**Conclusions:**

The data obtained in this study provided an overview of the viral community present in these rodent fecal samples, revealing some rodent-associated viruses closely related to known human or animal pathogens. Strengthening our understanding of unclassified viruses harbored by rodents present in the natural environment could provide scientific guidance for preventing and controlling new viral outbreaks that can spread via rodents.

## Background

Emerging infectious diseases have severely affected global public health [1]. Most of the newly emerging infectious diseases in the world originate from wild animals [2]. The spread of viruses may be affected by many factors, including the density of animals and the degree of contact with humans, among other factors [3]. Rodents are one of the animals in close contact with humans and have a high distribution density. Therefore, rodents, which are important reservoirs of viruses and are natural hosts of multiple viruses, are one of the wild animals that are extremely threatening to humans [4, 5]. Many viruses do not cause obvious clinical symptoms in rodents, but when the virus crosses to a human host, it may cause serious disease. For example, Hantavirus and Arenavirus are extremely harmful viruses to humans, but not to rodents.

With the continuous development of DNA sequencing technologies, many new viruses have been discovered in recent years[2, 4], including some pathogenic and non-pathogenic viruses that naturally exist in the host. With the continuous deepening of global research into viruses carried by rodents [6–9], a deep understanding of viruses carried by rodents has begun to be developed. Studies have found that most viruses related to public health have similar viruses in rodents. Such studies have also largely enriched the knowledge of virus diversity [6, 8–10] and provide a theoretical basis for the possible occurrence of zoonotic diseases in the future and tracking the origins of transmission. Many new viruses are reported continuously, indicating a large number of viruses that remain to be discovered. Therefore, virus composition and new virus mining in rodents from different geographic regions are greatly important for preventing and controlling new infectious diseases.

In this study, fecal pellets were collected from *Myodes rufocanus* (*MR*), *Apodemus peninsulae* (*AP*), *Apodemus agrarius* (*AA*), *Tamias sibiricus* (*TS*), *Sciurus vulgaris* (*SV*), and *Cricetulus triton* (*CT*), which are wild representatives of the mice forest areas of Hengdaohezi Town, Hailin City, and Heilongjiang Province. The feces from those species were used to assess the variety of viruses carried by forest rodents. Metagenomic next-generation sequencing analyses were performed to screen the viromes of the samples. Herein, we outlined the viral spectrum within these mouse samples and found a novel astrovirus. These data offer new clues for tracing the sources of important viral pathogens that can cause human and animal disease.

## Methods

### Sample processing

82 rodents were captured in the forested areas of Hengdaohezi Town (A, N 44°48′44″, E 129°02′04″) Hailin City, Heilongjiang Province from May 21 to August 22 (Summer Festival) in 2020 were selected as the research samples. The collected samples included 22 *Myodes rufocanus (MR),* 16 *Apodemus peninsulae (AP),* 15 *Apodemus agrarius (AA),* 11 *Tamias sibiricus (TS),* 10 *Sciurus vulgaris (SV),* and 8 *Cricetulus triton (CT).* A total of six samples from the same species were mixed.

Fecal pellets samples were homogenized, diluted in a ratio of 1:10 using PBS, made into a suspension, and vortexed for thorough mixing. The samples were then centrifuged at 2000 rpm for 10 min at 4 °C. Following this, the supernatants were transferred to a fresh tube and centrifuged for 10 min for the complete removal of cell debris, bacterial cells, and other impurities. The supernatants were filtered through a 0.22 μm syringe filter (Jet, Guangzhou, China) and concentrated. The filtrate was centrifuged in an SW55Ti rotor using a Beckman ultracentrifuge at 45,000 rpm for 2 h. The precipitates were re-suspended in PBS and passed through a 0.22 μm syringe filter. The samples were then stored at − 80 ℃ until subsequent analyses.

### Viral metagenomics analysis

Sequencing libraries were generated using the NEBNext® Ultra™DNA Library Prep Kit for Illumina (NEB, USA), following the manufacturer's recommendations. Index codes were added to attribute sequences to each sample. Briefly, the DNA samples were fragmented by sonication to a size of 300 bp, and the DNA fragments were then end-polished, A-tailed, and ligated with the full-length adaptor for Illumina sequencing to aid further PCR amplification.

Finally, PCR products were purified (AMPure XP system), and libraries were analyzed for size distribution using an Agilent2100 Bioanalyzer and quantified using real-time PCR. According to the manufacturer's instructions, the clustering of the index-coded samples was performed using a cBot Cluster Generation System. After cluster generation, the library preparations were sequenced on an Illumina HiSeq2500 platform, and paired-end reads were generated.

### Species annotation and analyses of abundance

Prinseq software (version 0.20.4) was used to assess the sample data quality, filter the low-quality and repetitive sequences and the rodent genome was the reference genome. Bowtie2 software was used to remove host DNA sequences, and Mira (v4.0.2) software was used to splice and assemble the sequences. The assembled and unassembled sequences were searched against the local NCBI virus database using the BLASTX and BLASTN tools in the BLAST + software package to obtain virus annotations. Geneious version 2019.2.1 was used to predict the ORF of the new annotated virus.

### Passage of fecal supernatants in BHK-21 cells

The BHK-21 was used to propagate the fecal suspensions from wild mice and detect viruses according to previously published methods [11, 12]. Briefly, the supernatants of homogenized fecal pellets were filtered through a 0.22 µm syringe filter and inoculated onto the BHK21 cells, followed by incubation for 2 h to allow for virus adsorption. After adding a fresh medium, the cells were incubated at 37 ℃ and monitored daily until 7 days postinfection to develop CPE. The infected cells were blindly transmitted three to six times until CPE appeared. The supernatant harvested from CPE-positive BHK-21 cells was further inoculated on BHK-21 cells to detect the presence of infectious viruses.

### Detection of astrovirus in CPE-positive cells

BHK-21 cells were inoculated with the supernatant collected from CPE-positive cells and incubated for 36 h. Real-time fluorescent quantitative reverse transcription PCR (RT-qPCR), immunofluorescence detection (IFA) were used to analyze the presence of astroviruses in the inoculated cells.For RT-qPCR analysis, primers that specifically amplify the target genes of astrovirus were designed based on the assembled contig sequences.

### Phylogenetic analysis

The sequences of reference strains with high similarity to the virus described in this study were downloaded from NCBI. A phylogenetic tree was constructed using MEGA software based on the neighbor-joining maximum composite likelihood method with 1,000 bootstrap replicates to analyze phylogenetic relationships.

### Statistical analysis

MetaStat was used to analyze the top 10 abundant taxonomic sequence tags of the three samples. The differences were considered to be statistically significant when the *P*-value was less than 0.05.

## Results

### Sequencing and quality control

DNA and RNA, extracted from fecal samples of 82 forest rodent intestinal contents samples collected in the forest area of Hengdaohezi Town Hailin City, Heilongjiang Province, were sequenced. The length of the insert size was 350 bp. Bases showing overlapping information and low mass, and bases that were not measured, were excluded. The total numbers of clean data obtained from the six samples were: 2,384.93 (*MR*), 2,090.33 (*AP*), 1,994.32 (*AA*), 2,391.77 (*TS*), 2,007.30 (*SV*), and 2,148.70 (*CT*).Sequencing data quality was distributed in the quality score Q20 to ensure a normal order of the subsequent advanced analysis. The clean sequence tags were subjected to redundancy processing using the Mothur software to obtain unique sequence tags. The percentages of effective sequences of the six samples were: 95.242%(*MR*), 93.561% (*AP*), 97.509% (*AA*), 97.258%(*TS*), 93.121% (*SV*), and 94.622% (*CT*). (Table 1).

**Table 1:** Fecal samples used for metagenomic analysis and data generation

Sample	Insert size (bp)	Seq strategy	Raw data	Clean data	Clean_Q20	Clean_Q30	Clean_GC (%)	Effective (%)
*Myodes rufocanus*	350	(150:150)	2,504.06	2,384.93	76.53	65.45	58.69	95.242
*Apodemus peninsulae*	350	(150:150)	2,234.18	2,090.33	75.94	64.76	57.70	93.561
*Apodemus agrarius*	350	(150:150)	2,045.27	1,994.32	87.12	78.68	52.56	97.509
*Tamias sibiricus*	350	(150:150)	2,459.21	2,391.77	83.63	73.78	52.87	97.258
*Sciurus vulgaris*	350	(150:150)	2,155.58	2,007.30	73.76	62.15	59.05	93.121
*Cricetulus triton*	350	(150:150)	2,270.82	2,148.70	78.59	67.93	55.53	94.622

### Viral communities in fecal samples, based on family-level classifications

**Fig.1:**
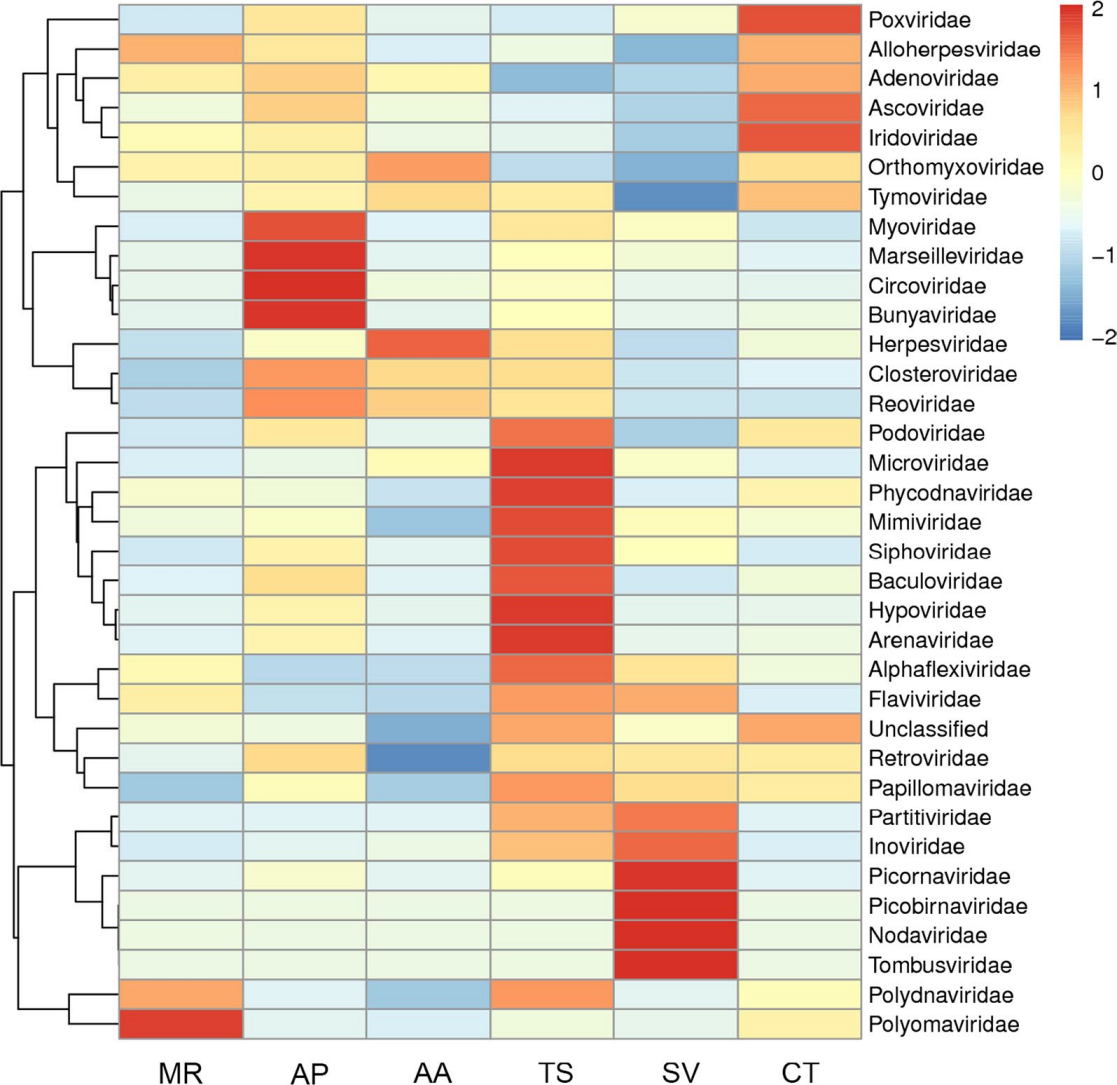
Heatmap based on the normalized sequence reads of 35 families of viruses in each sample. The horizontal axis was regarded as the sample name. Portrait-axis is species information. The diagram on the left side of the clustering tree is a species of tree. The below the clustering tree is a sample tree. The boxes colored from blue to red represent the metagenomic sequencing reads observed

**Fig. 2:**
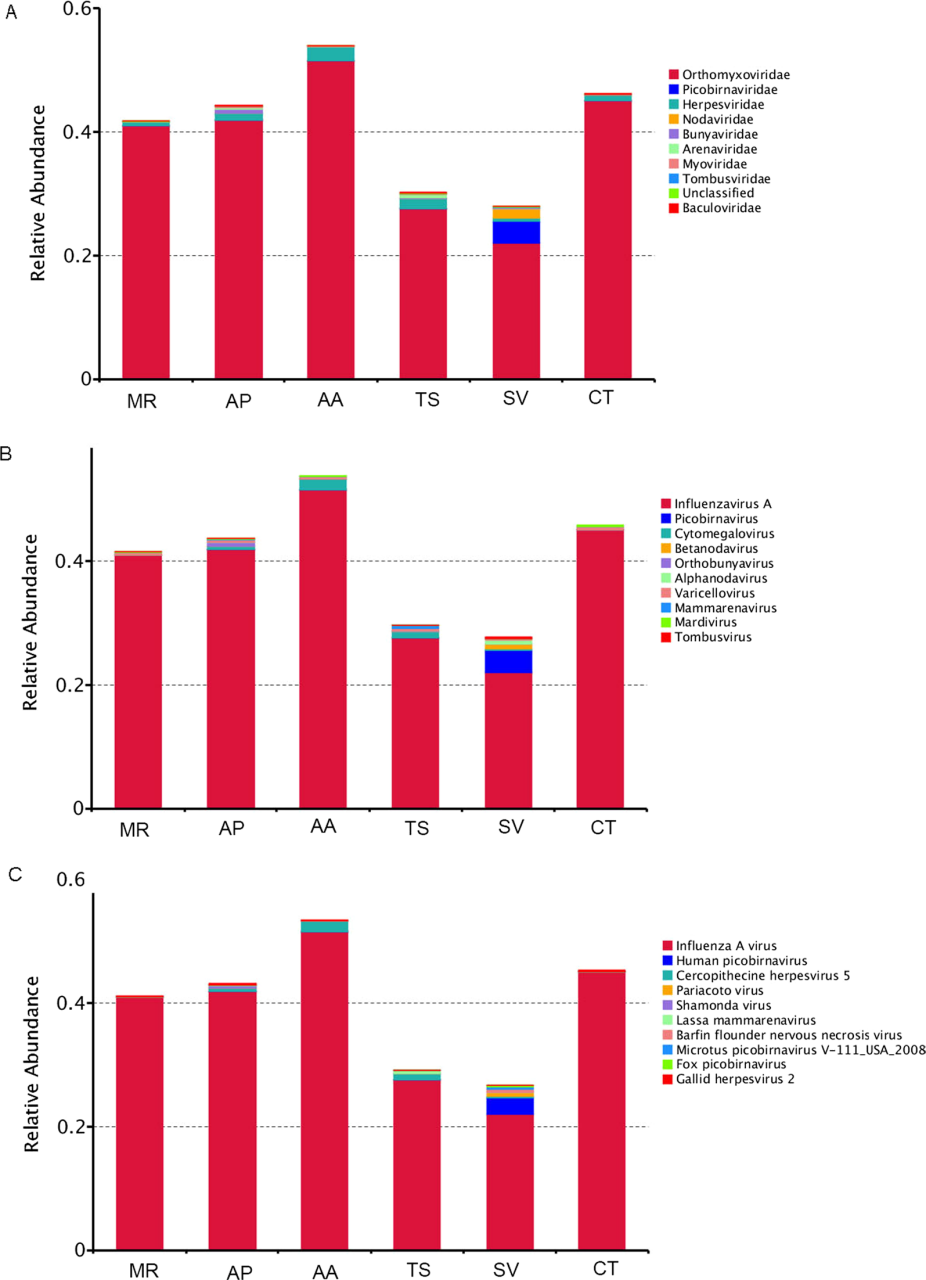
**A**, **B**, and ** C** represent the relative abundance of families, genera, and species, resectively, identified in each sample. The Y-axis represents the ratio annotation to a certain species, while the X-axis lists the sample names. The corresponding color blocks show the species category (legend on the right side)

Pre-treated clean data for all samples and the assembled sequence were compared to the reference genomes of viruses in the NCBI database using the BLASTX and BLASTN tools in the BLAST + software package to obtain the virus annotation results. In total, 82 families of mammalian viruses, plant viruses, phages, insect viruses, and fungal viruses were parsed. An overview of the reads of the top 35 families of viruses in each sample is shown in Fig. 1. In addition, an overview visualized presentation of the classification of families, genera, and species for each sample is shown in Fig. 2A–C.

- (1) Single-stranded RNA viruses(Orthomyxoviridae, Picobirnaviridae, Bunyaviridae, and Arenaviridae)
  The members of the family Orthomyxoviridae can cause cyclical pandemics throughout the world in various species[13].In this study, they are assigned to the genus Influenzavirus A and the species influenza A virus.The reads related to the family Orthomyxoviridae occupied the largest proportion of viruses. The percentage of this family of viruses in each sample was: 45.04% (*MR*), 51.57% (*AP*), 41.08% (*AA*), 41.9% (*TS*), 27.59% (*SV*), and 22.1% (*CT*) (Fig. 2A).
  The family members of Picobirnaviridae cause a wide variety of mucocutaneous, encephalic, cardiac, hepatic, neurological, and respiratory diseases invertebrate hosts [14]. The Picorbirnaviridae family viruses were found in all six samples. The viruses were assigned to the genus picobirnavirus, the species human picobirnavirus, Microtus picobirnavirus V-111_USA_2008, and fox picobirnavirus. It is worth mentioning that the human picobirnavirus occupied the larger proportion of viruses in the *SV* sample (2.64%) (Figure 2C).
  The family Bunyaviridae have strong infectivity, wide distribution, a high fatality rate, and can cause serious infectious diseases in humans and animals [15], Most of the members of this family, such as Rift valley fever virus, Crimean-Congo hemorrhagic fever virus, La Crosse encephalitis virus, and Hantavirus, cause deadly diseases in humans. The natural hosts for Hantavirus are rodents, and it can cause hemorrhagic kidney fever. The virus was present in all samples of the six forest rodents. Among these, the abundance in fecal of *SV* (3.49%) was higher than that in the other samples. The viruses of this family were assigned to the genus, Orthobunyavirus, and the species, Shamonda virus (Fig. 2B, C).
  Arenaviridae is an enveloped RNA virus found worldwide. The Lassa fever virus, Junin virus, and Machupo virus can cause severe diseases with a high mortality rate[16]. Thus, the prevalence of infectious diseases is closely related to the local dynamic distribution of rodents. In this study, the virus was detected in the fecal of all six species of mice.The viruses in this family are assigned to the genus, Mammarenavirus, and the species, Lassa mammarenavirus.
- (2) DNA viruses (Herpesviridae)
  Viruses of the Herpesviridae family are enveloped, double-strand DNA viruses, divided into three genera based on phylogenetic clustering: *α*-herpesvirus, *β*-herpesvirus, and *ɣ*-herpesvirus [17]. This family was detected in fecal samples of all six species of rodent. The viruses in this family were assigned to the genera, Cytomegalovirus, Varicellovirus, Mardivirus, and the species, Cercopithecine herpesvirus 5 and Gallid herpesvirus 2, respectively (Fig. 2B, C).
- (3) Other rare viruses (Nodaviridae, Baculoviridae, Tombusviridae, Myoviridae)
  Insect viruses (Nodaviridae, Baculoviridae), plant viruses (Tombusviridae), and phages (Myoviridae) were identified in the fecal samples. The viruses in the family of Nodaviridae were assigned to the genera, Alphanodavirus and Betanodavirus, and the species, Pariacoto virus and Barfin flounder nervous necrosis virus, respectively. The viruses in the family of Tombusviridae were assigned to the genera, Tombusvirus, and no virus in the family of Tombusviridae was assigned to the species among the top 10 most widely distributed. It is worth mentioning that *AA* and *CT* did not contain any members from Tombusviridae.No virus in the family of Baculoviridae and Myoviridae was assigned to the genera, and species among the top 10 most widely distributed.
- (4) Unclassified viruses
  Currently, there is little information about unclassified viruses and their evolution in forest rodents. In our data, many reads are classified as "unclassified virus sequences" in all samples, likely to be previously unidentified viruses that have not been studied. The identification and characterization of these unclassified viruses will provide insight into the evolutionary histories of other clinically important viruses, as well as the genetic basis behind their infectivity and virulence in humans and other animals. Such information is important for the development of future treatment options and vaccine research (Fig. 2A).

### Phylogenetic analysis of zoonotic

**Fig. 3:**
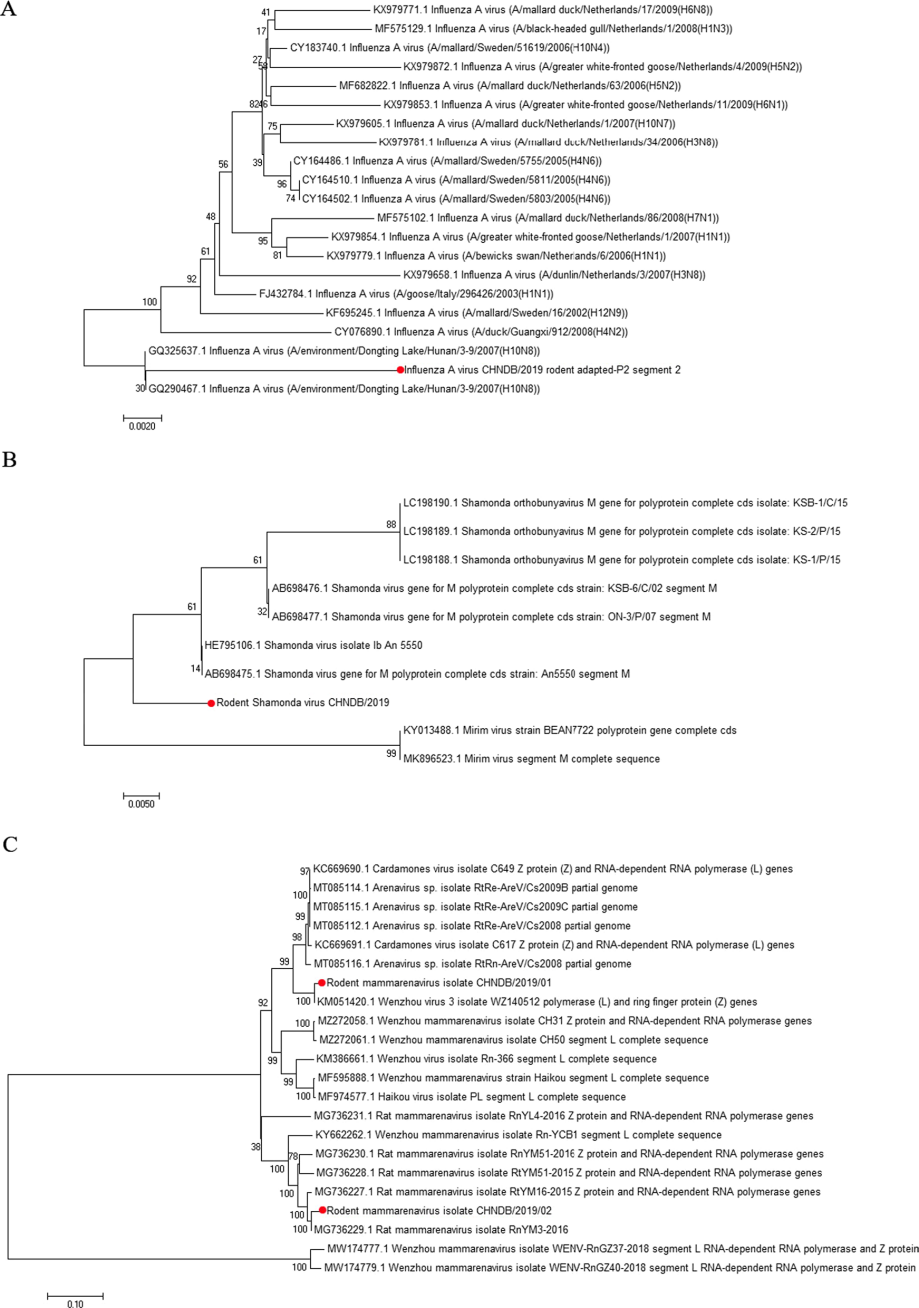
Phylogenetic relationships between the spliced important zoonotic described in this study in individual rodents and other isolates **A**, Influenzavirus A **B**, Shamonda virus, and **C**, Mammarenavirus. All three trees were constructed using the neighbor-joining maximum composite likelihood method with 1000 bootstrap replicates. The viruses this study are marked with black circle; the scale bar indicates genetic distance

- (1) Influenza virus A
  The results of bioinformatics analysis showed that a consistent sequence of Influenza virus A was obtained in six voles, and a PB1 fragment of 2341 bp was obtained after splicing, which was named Rodent Influenza virus A CHNDB/2019. The results of the sequence alignment analysis revealed that this strain had 96.79–98.72% nucleotide sequence similarity with virus strains found in environments or other organisms; among them, the similarity to GQ325637.1 Influenza A virus (A/environment/Dongting Lake/Hunan/3–9/2007(H10N8)) mouse-adapted strain was the highest. The results of the phylogenetic tree of the PB1 segment showed that the virus was on the same tree branch as A/environment/Dongting Lake/Hunan/3–9/2007(H10N8), and the genetic distance between them was the closest (Fig. 3A).
- (2) Shamonda virus
  The results of bioinformatics analysis showed that a completely identical splicing sequence of Shamonda virus was obtained in six different species of rodents. An M fragment of 4314 bp was obtained through splicing, named Rodent Shamonda virus CHNDB/2019. Due to the relatively few Shamonda virus sequences published by NCBI at present, the homology alignment results showed that very few sequences were highly homologous to the genome of this virus. The nucleotide sequence similarity with Shamonda virus isolate Ib An 5550 was the highest (96.38%), and the similarity with other virus strains was 86.21%–96.20%. Phylogenetic tree analysis showed that this virus's M gene has low homology to the genes of known viruses (Fig. 3B).
- (3) Mammarenavirus
  Samples from *AA*, *TS*, *SV*, *CT*, and *AP* rodents were sequenced, and a consistent spliced Lassa mammarenavirus L fragment, with a sequence length of 7178 bp, named Rodent mammarenavirus CHNDB/2019/01, was obtained. On the other hand, a 7121 bp fragment was obtained from the *MR* rodent, by sequencing Fragment L; it was named Rodent mammarenavirus CHNDB/2019/02. Sequence analysis showed that the nucleotide sequence similarity between the Rodent mammarenavirus CHNDB/2019/01 L fragment and the Wenzhou Mammarenavirus isolate MYR-039 was the highest at 98.59%, while that between the Rodent mammarenavirus CHNDB/2019/02 L fragment and the rat mammarenavirus isolate RnYM3-2016 was the highest at 97.89%. Phylogenetic tree analysis of L fragment was consistent with the sequence similarity results. Rodent mammarenavirus CHNDB/2019/01 and Wenzhou Mammarenavirus MYR-039 isolate were clustered on one branch, while Rodent mammarenavirus CHNDB/2019/01 and rat mammarenavirus isolate RnYM3-2016 were clustered on another branch (Fig. 3C).

### Genome features of the novel astrovirus

Using mNGS, we identified a novel astrovirus from the feces of *Myodes rufocanus*, which was tentatively named Rodent Astrovirus CHNDB/2019. Rapid Annotation using Subsystem Technology (RAST, http://rast.nmpdr.org) was used to annotate the complete genome sequence. The results revealed that the Rodent Astrovirus CHNDB/2019 genome consists of 6192 nt and contains two open reading frames: ORF1 encodes a polyprotein located at nucleotide (nt) positions 35-3750, and ORF2 was located at nt positions 3719 -6178 in *Myodes rufocanus*.

### Phylogenetic analysis

**Fig. 4:**
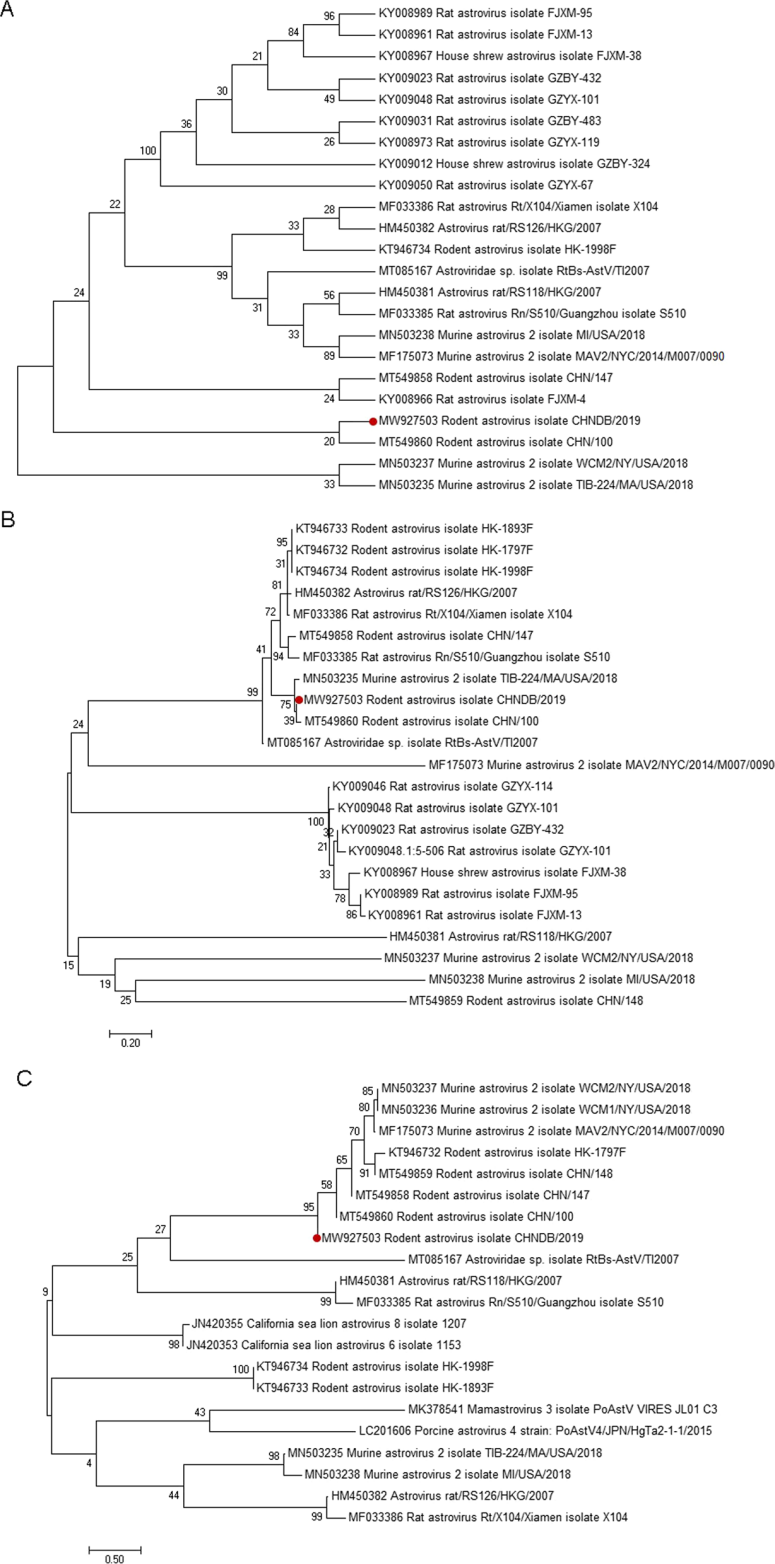
Phylogenetic relationships between the novel rodent astrovirus described in this study and other rodent astrovirus isolates **A**, concatenated ORFs, **B **ORF1ab, and **C** ORF2 sequences. All three trees were constructed using the neighbor-joining maximum composite likelihood method with 1000 bootstrap replicates. The viruses found in this study are marked with black circle; the scale bar indicates genetic distance

The phylogenetic analysis of the complete genome sequence showed that ORF1 (Fig. 4B), ORF2 (Fig. 4C), and the complete genome (Fig. 4A) had the highest similarity to the rodent astrovirus isolate, CHN/100, which was isolated from Jiaxiang County, Shandong Province, and the genetic distance between them is the closest. The nt sequence similarity between the entire genome and CHN/100 was 84.39%,while between the entire genome and other rodent astroviruses was 78.08%–67.74%. The nucleotide sequence similarity between ORF1 and orf1b of Rodent Astrovirus isolate CHN/100 was 86.2%, and the amino acid similarity was 74.45%. The greatest similarity between OFR2 and orf2 of the rodent astrovirus isolates CHN/100 was 81.27%, while the amino acid sequence similarity was 63.61%. Therefore, it was clear that the virus was a rodent astrovirus (GenBank Submission Number, MW927503).

### Infectivity of astroviruses in BHK cells

**Fig. 5:**
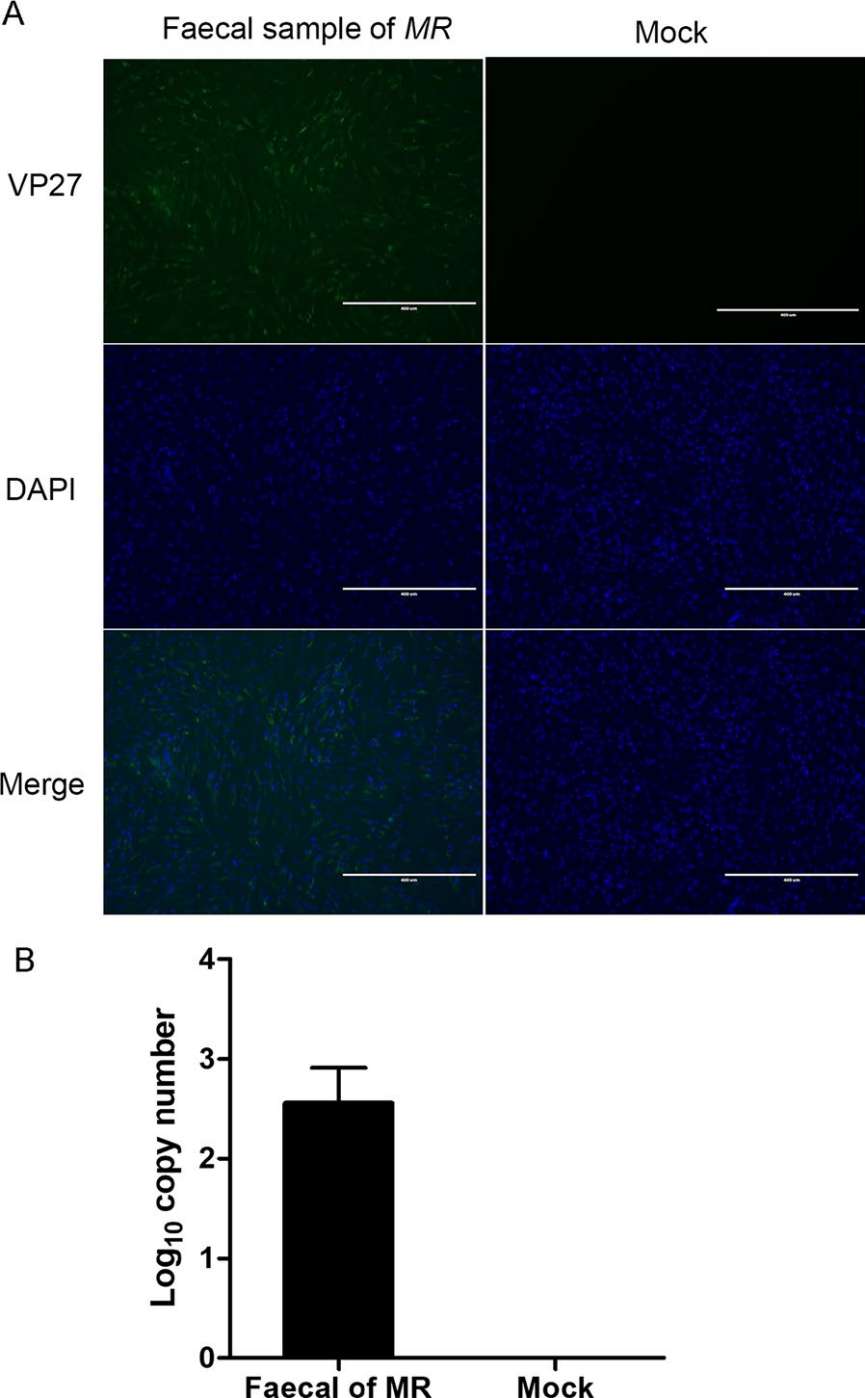
The new rodent astrovirus was detected in BHK-21 cells. BHK-21 cells were inoculated with supernatants collected from CPE-positive cells inoculated with Myodes rufocanus fecal samples for 36 h. The astrovirus in the samples was detected by IFA **A** and RT-qPCR **B**. The nucleus was stained with DAPI

The fecal samples containing the new astroviruses were inoculated into BHK-21 cells to detect the presence of infectious astroviruses. After four to five blind passages, CPE appeared in BHK cells. IFA and RT-qPCR were used to analyze the presence of astroviruses, which could be detected in the inoculated cells. IFA (Fig. 5A) confirmed the presence of antibodies specific to the astrovirus capsid spike protein, VP27. In addition, whether there is astrovirus in the inoculated BHK-21 cells. RT-qPCR was performed using astrovirus ORF1 gene-specific primers ORF1-F (5′-CAGTCCTTGGGATTTCTC-3′); ORF1-R (5′-TATTCTTTCGCACCATTAG-3′) (Fig. 5B).

## Discussion

Currently, there have been some reports about the metagenomic investigations of the viruses carried by rodents. Duan et al. collected the intestinal contents of 99 *Rattus norvegicus* rats and *Apodemus agrarius* mice in Jiaxiang County, Shandong Province, and studied their viral compositions via high-throughput sequencing. The study also found that multiple strains of astrovirus were circulating among rodents in this geographic area at the same time, indicating the extremely rich genetic diversity[18].Vandegrift et al. analyzed the serum viromes of 978 free-range *P. leucopus* mice captured in Pennsylvania and discovered multiple new viruses from 26 different families, among which there was a highly divergent segmented flavivirus [19].Williams et al. analyzed the viruses from mouse feces in seven residential locations in Manhattan, Queens, Brooklyn, and Bronx, New York City, New York, within the same year. Unbiased high-throughput sequencing revealed 36 viruses from 18 families and 21 genera, including at least six new viruses and three new genera[8]. In this study, fecal samples from forest rodents in Northeast China were sequenced using metagenomics, and an abundance of virome information was acquired. 82 families of mammalian viruses, plant viruses, insect viruses, and phages were detected. Among the top 10 most abundant families were the RNA viruses, Orthomyxoviridae, Picornaviridae, Bunyaviridae, and Arenaviridae; the DNA virus, Herpesviridae; the insect viruses, Nodaviridae and Baculoviridae; the plant virus, Tombusviridae; and the phage, Myoviridae.

Influenza virus A, Shamonda virus, and Mammarenavirus are all single-stranded RNA zoonotic viruses that are found in voles [13–15]. In this study, we selected important zoonotics from individual rodents for sequencing and further evolutionary analysis. With the change in human habitation, forest rodent habitats are often close to human settlements and they have frequent contact.The high frequency of human contact with forest rodents have greatly increased the threat of zoonotic pathogens to public health and safety [4, 5]. Therefore, a better understanding of pathogen sequence characteristics is of great significance for tracking the origins of transmission and preventing human infectious diseases.This study selection of important zoonotic in individual rodents for further sequence and evolutionary analysis.

Astrovirus is a type of virus that infects mammals and poultry and has a wide host range. Astrovirus infection in humans can cause gastrointestinal diseases, and there have been a few cases of neurological symptoms [20–22]. Many new animal astroviruses have been discovered in recent years [9], and such rich genetic diversity among mammalian astroviruses may be caused by the cross-species transmission in wild animals, domestic animals, or humans [20]. This study also identified new viruses. A new astrovirus was identified through sequence splicing, software prediction, evolutionary tree analysis, and experimental verification, and the sequence was submitted to NCBI. There likely are more than the newly identified virus in the samples. Due to the limited sample size in this study, there may be deviations in these rodents that carry the virus. Therefore, further research will be performed to increase the sample size and expand the range of sampling sites.

## Conclusions

In this study, fecal samples from six species of forest rodents in Northeast China were sequenced using metagenomics, and an abundance of virome information was acquired.The top 10 most abundant families were Orthomyxoviridae, Picornaviridae, Bunyaviridae, Arenaviridae, Herpesviridae, Nodaviridae, Baculoviridae, Tombusviridae, Myoviridae and Unclassified viruses. In addition, selection of important zoonotic in individual rodents for further sequence and evolutionary analysis. A new strain of astrovirus has been discovered, with an ORF genome arrangement comparable to other rodent astroviruses. It has the highest similarity with the rodent astrovirus isolate CHN/100. The metagenomics approach can greatly improve our understanding of the diversity of viruses in mice. This study analyzed the composition and abundance of virus genomes in fecal from six species of rodents using metagenomics technology. This strategy could be extended to other wild or livestock samples worldwide, ultimately increasing our knowledge of the viral population and ecological community, which would help minimize the impact of potential wildlife-associated viruses on public health by providing meaningful basic data.

## Data Availability

The datasets analyzed in the current study are available from the corresponding author on reasonable request.

## Abbreviations

ORF: Open reading frame
BHK21: Baby-hamster kidney cell line
CPE: Cytopathic effects
